# Is attachment style in early childhood associated with mental health difficulties in late adolescence?

**DOI:** 10.1192/bjo.2021.98

**Published:** 2021-06-18

**Authors:** Philippa Clery, Angela Rowe, Marcus Munafò, Liam Mahedy

**Affiliations:** University of Bristol

## Abstract

**Aims:**

Identifying factors that contribute to mental health difficulties in young people as early in life as possible are needed to inform prevention strategies. One area of interest is attachment. Although existing research has suggested an association between insecure attachment styles and mental health difficulties, these studies often have small sample sizes, use cross-sectional designs, and measure attachment as a discrete variable at a single point or use romantic relationship attachment as a proxy for childhood attachment. It is also unclear whether these associations persist into late adolescence. In this large prospective study we aimed to determine whether an insecure attachment style measured at repeated points in early childhood, is associated with depression and self-harm at 18 years.

**Method:**

We used data from the Avon Longitudinal Study of Parents and Children cohort. Mothers completed attachment related questionnaires when their child was 18, 30, and 42 months old. Offspring depression and lifetime self-harm was assessed at 18 years in clinic using the Clinical Interview Schedule-Revised. Attachment was derived as a continuous latent variable in a structural equation modelling framework. Logistic regression was performed on participants with complete attachment data (n = 7032) to examine the association between attachment style and depression and self-harm, with adjustment for potential confounders. Differential dropout was accounted for using multiple imputation.

**Result:**

We found some evidence for an association between a more insecure attachment style in childhood, and a diagnosis of depression and life-time self-harm at age 18. In the fully adjusted imputed model, a one standard deviation increase in insecure attachment was associated with a 13% increase in the odds of depression (OR = 1.13; 95%CI = 1.00 to 1.27) and a 14% increase in the odds of self-harm at age 18 (OR = 1.14; 95%CI = 1.02 to 1.25), for children who had more insecure attachment in early childhood, compared with children who had more secure attachment.

**Conclusion:**

This is the largest longitudinal study to examine the prospective association between childhood attachment and depression and self-harm in late adolescence. Our findings strengthen the evidence suggesting that a childhood insecure attachment style is associated with mental health difficulties in late adolescence. Policies and interventions to support parenting behaviours that foster the development of secure attachment styles, or attachment-based therapies to improve attachment quality, could help reduce depression and self-harm in adolescence/young adulthood.

Philippa Clery is supported by the Elizabeth Blackwell Institute for Health Research at the University of Bristol and the Wellcome Trust Institutional Strategic Support Fund.

